# Alexithymia as a mediator of the associations between child maltreatment and internalizing and externalizing behaviors in adolescence

**DOI:** 10.1038/s41598-024-56909-2

**Published:** 2024-03-16

**Authors:** Catherine Hamel, Christopher Rodrigue, Camille Clermont, Martine Hébert, Linda Paquette, Jacinthe Dion

**Affiliations:** 1 Département de Psychologie, Université de Montréal, Montréal, H2V 2S9 Canada; 2 Research Centre On Intimate Relationship Problems and Sexual Abuse (CRIPCAS), Université de Montréal, Montréal, H2V 2S9 Canada; 3 École de Psychologie, Université Laval, Québec, G1V 0A6 Canada; 4 Département de Sexologie, Université du Québec À Montréal, Montréal, H2L 4Y2 Canada; 5 Département Des Sciences de La Santé, Université du Québec À Chicoutimi, Chicoutimi, G7H 2B1 Canada; 6 Département de Psychologie, Université du Québec À Trois-Rivières, Trois-Rivières, G9A 5H7 Canada

**Keywords:** Risk factors, Signs and symptoms

## Abstract

Child maltreatment is a global concern that profoundly affects individuals throughout their lives. This study investigated the relationships between various forms of child maltreatment and behavior problems involving internalization and externalization during adolescence. Data obtained from a diverse sample of 1802 Canadians aged 14–18 years was used to examine the mediating role of alexithymia—a difficulty in recognizing and expressing emotions—in these associations. Results indicated that adolescents who experienced sexual abuse, emotional abuse, and exposure to intimate partner violence (IPV) in their childhood exhibited higher levels of alexithymia, which was correlated with elevated levels of both internalizing and externalizing problems. Physical abuse and parental neglect were only associated with externalizing problems. Gender differences also emerged, with gender-diverse adolescents reporting a higher prevalence of maltreatment, alexithymia, and behavior problems compared with their peers. However, alexithymia’s mediating role was consistent across genders. Overall, this study highlights the intricate relationships between child maltreatment, alexithymia, and adolescent behavior problems. The findings of this study how different forms of child maltreatment significantly shape behavioral outcomes and indicate the importance of interventions in enhancing emotional awareness and expression in adolescents with a childhood history of maltreatment.

## Introduction

Child maltreatment is a major issue worldwide^1^. In 2014, findings from a national survey revealed that one-third of Canadian adults reported having suffered abuse during childhood— whether sexual or physical abuse or exposure to intimate partner violence (IPV)^2^. Numerous longitudinal studies have shown that such adverse childhood experiences can lead to significant adaptation difficulties, including internalizing (e.g., depression, anxiety, somatization) and externalizing behavior problems (e.g., aggression, disobedient behaviors)^3–6^ that persist into adolescence^3,4^. On the one hand, internalizing behaviors have been found to be common among children and to increase in adolescence^7,8^. Among adolescents, recent research on internalizing problems found associations with suicidality^9^ and poor health outcomes^10^. On the other hand, longitudinal studies have shown that children with externalizing behavior problems at an early age are more likely to drop out of high school^11^, develop conduct disorders and antisocial behavior in adolescence^12^, and have substance abuse problems in the long term^13^.

Despite abundant literature on child maltreatment and its associations with behavior problems, significant research gaps remain, including the need to investigate the specific influence of each maltreatment form (e.g., sexual abuse, physical abuse, emotional abuse, parental neglect, and exposure to IPV)^14^ in relation to internalizing and externalizing behavior problems^15^. Indeed, some studies have suggested that certain maltreatment forms are differentially associated with behavioral problems. For example, emotional abuse has been associated with internalizing and externalizing problems, whereas physical abuse has been associated only with externalizing problems^16^. Sexual and physical abuse have also been associated with increased risk of both internalizing and externalizing problems^15^.

Another significant gap involves the scarcity of studies documenting possible mechanisms in associations between child maltreatment and subsequent difficulties. Negative outcomes generated by child maltreatment indicate the need to understand better these associations’ underlying mechanisms so as to identify potentially effective interventions. In the broader spectrum of emotional regulation abilities, alexithymia, which represents a more refined and distinct form of emotion dysregulation^17^, may be of particular interest. Indeed, according to Taylor and Bagby^18^, child maltreatment is a known etiological factor in the development of alexithymia, which refers to difficulties in identifying, expressing, and describing one’s emotions and by limited ability to recognize and understand others’ emotions^19^. Studies have shown that when compared to individuals without a history of maltreatment, victims of childhood maltreatment report higher levels of alexithymia^20^. Based on Ditzer et al.’s meta-analysis^21^, children growing up in abusive environments such as those characterized by maltreatment do not properly learn to regulate their emotions or tolerate negative affects. They also lack modeling and reinforcement of appropriate coping strategies and emotional expressivity^20,22^. In such environments, expression of negative emotions is often invalidated or punished, thus interfering with normal development of identification and expression of emotions^22^ and causing difficulties in processing emotional responses to negative emotional stimuli^23^. Previous studies have indicated that the ability to emotionally regulate may be a key factor in explaining the relationships between different forms of child maltreatment and internalizing and externalizing behaviors^9,24^. Traumatic events, including maltreatment, may interfere with the ability to emotionally regulate and thus may lead to difficulties in adaptation. Among children and adolescents, in fact, alexithymia has been associated with both internalizing and externalizing behavior problems^25,26^. Taylor et al.^27^ hypothesized that individuals experiencing alexithymia may have limited capacity to process their emotions cognitively, leading them to adopt maladaptive behaviors (such as internalizing and externalizing problems) as strategies for coping with emotional distress. Therefore, alexithymia appears to be a potential mediator in associations between child maltreatment and internalizing and externalizing behavior problems.

In a recent meta-analysis, Khan and Jaffee^28^ highlighted alexithymia’s mediating role in associations between childhood maltreatment and several psychological disorders across lifespan. Among children, alexithymia has been found to mediate relationships between sexual abuse and both internalizing and externalizing behavior problems^29^. Moreover, low emotional awareness (i.e., a construct of alexithymia) mediated associations between childhood violence exposure and psychopathology (e.g., internalizing and externalizing problems)^30^. In adulthood, alexithymia has been identified as a mediator of relationships between child maltreatment and sexual risk taking^31^, impulsivity ^22^, and self-injurious behaviors^32^, and suicidal behaviors^33^. It has also been found to mediate associations between different forms of maltreatment (i.e., sexual, physical, and emotional abuse) and disordered eating^34,35^, and between emotional neglect and symptoms of depression, anxiety, and loneliness ^20^. The few studies exploring this issue with adolescent samples have suggested that alexithymia mediated the relationship between child sexual abuse and psychological distress^36^. Child maltreatment was also associated with emotional processing difficulties, which were linked to alexithymia, and subsequently, alexithymia was correlated with the severity of psychological symptoms^37^.

To our knowledge, no study has assessed alexithymia’s mediating role in the relationship between various forms of maltreatment and adolescents’ internalizing and externalizing behavior problems. Instead, the bulk of research has focused mainly on adults, whose results might not generalize to adolescence, a dynamic and critical phase marked by heightened emotional responses to environmental stimuli and great changes at social, physical, sexual, and intellectual levels^38^. During this developmental period, many adolescents experience significant improvement in emotion regulation abilities^39^. Nevertheless, for others, adolescence becomes a turning point or exacerbates psychopathological issues marked by difficulties in emotion regulation^39^. Experiencing internalizing and externalizing problems during adolescence can also lead to maladaptive development in adulthood^40^.

### Study objective

In sum, while associations between child maltreatment, behavior problems, and alexithymia have been demonstrated, significant gaps remain in the literature as to whether, during adolescence, different forms of earlier child-maltreatment influence development of alexithymia and both internalizing and externalizing problems. Therefore, this study tested an integrated model in which alexithymia is proposed as a mediator of links between various forms of child maltreatment (sexual abuse, physical abuse, emotional abuse, parental neglect, and exposure to IPV) and internalizing and externalizing behavior problems among adolescents. Based on previous studies, we expected that each form of child maltreatment would be positively associated with internalizing and externalizing behavior problems. We also hypothesized that these associations would be mediated by alexithymia. More specifically, higher levels of various forms of maltreatment were expected to be associated with higher levels of alexithymia, which in turn would be associated with higher levels of internalizing and externalizing behavior problems. Because previous alexithymia research has produced mixed results regarding possible gender differences—with some identifying a higher degree of alexithymia in boys^41^ and others in girls^42^, while yet others have identified no difference^20,36^—we also tested separate models for girls, boys, and gender-diverse adolescents.

## Material and methods

### Participants and procedure

#### Participants

Our sample included 1802 adolescents from 14 to 18 years old (*M* = 14.74; *SD* = 0.84). Most participants identified with the Québécois culture (67.2%), and 32.8% reported other cultural identities, including Canadian, American, East European, West European, African, Asian, Middle Eastern, Latin/South American, and Caribbean. Regarding gender identity, 55.9% of participants identified as *boy* (*n* = 1007), 42.5% as *girl* (*n* = 765), 1.4% as *gender-diverse* (*nonbinary, gender fluid, two-spirit* or *other*; *n* = 26), and 0.2% declined to answer (*n* = 4).

#### Procedure

Data were collected as part of a Canadian study on sport participation and resilience from October to December 2019. Recruitment was conducted in two distinct phases—first school and then participant. To ensure sample diversity, this study recruited ninth- and tenth-grade adolescents from differing socioeconomic backgrounds and from urban, semi-urban, and rural Canadian schools.

After the research coordinator detailed the research project to seven schools, six agreed to participate; one school refused due to a separate ongoing project. The selection criteria were as follows: (i) attending ninth grade; (ii) being at least 14 years old; and (iii) having no intellectual disability. In Quebec, consenting adolescents aged 14 years and older can participate in research without parental consent. In particular, according to the Civil Code of Quebec (chapter CCQ-1991, article 21), “Consent to research that could interfere with the integrity of a minor may be given by the person having parental authority or the tutor. A minor 14 years of age or over, however, may give consent alone if, in the opinion of the competent research ethics committee, the research involves only minimal risk and the circumstances justify it”^43^. Our research involved only minimal risk; thus, we obtained ethical approval to conduct our research. Omitting the need for parental consent can ensure the safety of students involved in the study and prevent sampling bias that could distort the results^44^. Before participation, the participants received detailed information about the research and provided informed consent. Of 1900 adolescents invited to participate, 1857 accepted (a 97.4% participation rate). In their classrooms, participants completed an anonymous self-report survey (Qualtrics Research Suite) on electronic tablets provided by research assistants. The survey included three attention-testing questions and was completed in an average of 40 min. Of 1857 responses, 11 were excluded for ineligibility (younger than 14); 40 were excluded because respondents did not provide a correct answer to all three attention-testing questions; and four were excluded because of inconsistent response patterns. After exclusions, the sample numbered 1802. Study participants were compensated with a reusable water bottle and entry into a raffle for a gift certificate. The research procedure was approved by the Institutional Review Board of Université du Québec à Chicoutimi (protocol code 2019–221 and date of approval: 2019–09-26) and performed according to the Declaration of Helsinki.

### Measures

#### Sociodemographics

Participants provided demographic information, including their age, culture, and language of origin. Based on prior recommendations, gender identity was assessed with the following item based on prior recommendations: *“What gender or gender identity do you identify with”*^45^*?* Response options included “*male*” or “*female*”; *indigenous or other cultural gender minority identity (e.g., two-spirit)*; *nonbinary, gender fluid, or something else (e.g., genderqueer)*; and *other* (with specification). For this study’s purpose, a categorical variable was computed to distinguish boys, girls, and gender-diverse individuals. Participants who reported *indigenous or other cultural gender minority identity (e.g., two-spirit); nonbinary, gender fluid, or something else (e.g., genderqueer)*; and *other* were considered gender-diverse individuals.

#### Child maltreatment

Five forms of child maltreatment were assessed: sexual, emotional, and physical abuse; parental neglect; and exposure to IPV. First, sexual abuse was measured using three dichotomous items (*yes/no*) from a subscale of the Early Trauma Inventory Self Report–Short Form (ETISR-SF)^46^, and adapted in a large Canadian survey^47^. For the current study, this questionnaire evinced adequate internal consistency (α = 0.67). Second, physical abuse was measured by one item from the Longitudinal Study of Adolescent Health^48^:*“During your childhood, how often did a parent/guardian push you, shove you, hit you or twist your arm”?* Third, a further two items of the ETISR-SF^46^ were used to assess emotional abuse during childhood: *“How often did a parent/guardian treat you with coldness, indifference, or in a way that you felt unloved”?* and “*How often did a parent/guardian ridicule or humiliate you”*^47^*?* In our sample, these items correlated positively and moderately (*r* = 0.44). Fourth, parental neglect was assessed by two items from the Neglect subscale of the Intimate Partner Violence questionnaire (IPV-Neglect)^48^: *“During your childhood, how often did a parent/guardian not take care of your basic needs, such as keeping you clean or providing food or adequate clothing”?* and *“How often did your parents/guardians leave you alone when an adult should have been with you”?* These items correlated positively and moderately (*r* = 0.32). Fifth, exposure to IPV was measured by three items adapted from the Revised Conflict Tactics Scale^49^ (e.g., *“During my life, I have seen one of my parents/guardians do this to my other parent/guardian: push, shove, slap, twist the arm, throw an object that could hurt …”*). Cronbach’s alpha showed acceptable internal reliability for these items (α = 0.74). Given these variables’ differences in measurement, all indicators were dichotomized (i.e., 0 = *no experience of child maltreatment*; 1 = *one or more experiences of child maltreatment*).

#### Alexithymia

Besides the scales listed above, participants completed an abbreviated four-item version^36^ of the Toronto Alexithymia Scale (TAS-20)^50^, which is widely used to assess alexithymia. This abbreviated version was selected in response to previous research indicating that a two-factor structure (difficulty in identifying and describing feelings), excluding the externally oriented thinking factor, had better model fit and increased the homogeneity of the alexithymia concept, particularly for adolescent populations^51,52^. The items included the following: (1) “It is difficult for me to find the right words for my feelings”; (2) “When I am upset, I do not know if I am sad, if I am frightened, or if angry”; (3) “I have feelings that I can’t quite identify”; and (4) “I am often confused about what emotion I am feeling.” Participants responded on a 5-point Likert-type scale (0 = *False*; 1 = *Rather false*; 2 = *Sometimes false, sometimes true*; 3 = *Rather true*; and 4 = *True*). Total scores ranged from 0 to 16, with higher scores indicating greater difficulty in identifying and expressing emotions. For the current study, the abbreviated TAS-20 demonstrated good internal consistency (α = 0.88).

#### Internalizing and externalizing behavior problems

Behavior problems were assessed using a validated version^53,54^ of two subscales of the Strengths and Difficulties Questionnaire (SDQ), a measure estimating the presence of behaviors that reflect emotional difficulties, for instance, aggression, isolation, and anxiety^55^. The *Emotional symptoms* subscale assesses internalizing behaviors, defined as behaviors directed inward, such as anxiety or depression symptoms^56^. The *Conduct problems* subscale assesses externalizing behaviors directed outward toward the social environment^57^, such as bullying, lying, and stealing. Each subscale contained 10 items (five each for internalizing and externalizing behaviors) answered on a 3-point Likert-type scale (0 = *Never*; 1 = *Somewhat true*; and 2 = *Certainly true*). Each subscale’s total scores ranged from 0 to 10, with higher scores indicating greater frequency of the behaviors. The *Emotional symptoms* subscale showed good internal consistency (α = 0.81). However, internal consistency was quite low for the *Conduct problems* subscale, and one item (the reverse score of “*I usually do as I am told*”) was removed from the original subscale to increase consistency (α = 0.58). Therefore, total scores for the *Conduct problems* subscale ranged from 0 to 8.

### Statistical analyses

To assess distribution between the study’s variables, descriptive statistics, Pearson correlations (including point-biserial correlations for relationships between dichotomous and continuous variables), Cronbach’s alphas, chi-square tests, and group comparisons were performed using SPSS version 28. Chi-Square tests (including Bonferroni post hoc tests) and univariate analysis of variances (ANOVAs) were also conducted to determine gender differences. In ANOVAs, Welch’s ANOVA F test with Bonferroni post hoc tests for group comparisons were used since the homogeneity of variance assumption was violated. M*plus* 8^58^ was used to build a structural equation model (SEM) to test the integrated model suggesting that alexithymia mediated relationships between various forms of child maltreatment (i.e., sexual, physical, and emotional abuse; parental neglect; and exposure to IPV) and internalizing and externalizing behavior problems. Before structural equation modeling, assumptions of multivariate analyses were verified. Because all assumptions, apart from normality, were met, we used the robust maximum-likelihood (MLR) estimator that can provide robust fit indices and standard errors with non-normal distributions. First, the model was examined using the whole sample (Model 1). Nonsignificant paths were then removed, providing model fit indices (Model 2). Multi-group SEM was then used to test whether forms of child maltreatment and alexithymia had similar associations with internalizing and externalizing behaviors in boys, girls, and gender-diverse individuals (Model 3). The difference test was pushed forward to ensure its significance, and path coefficients between variables of interest were constrained to be equal across groups (Model 4). Models 3 and 4 were then compared, and changes in chi-square, CFI, TLI, and RMSEA were inspected. Constrained and freely estimated models differed significantly in boys, girls, and gender-diverse individuals when those criteria were met: specifically, a significant corrected chi-square difference test, significant decreases in CFI and TLI (ΔCFI ≤ 0.010; ΔTLI ≤ 0.010), and significant increases in RMSEA (ΔRMSEA ≤ 0.015)^59,60^. Additionally, to determine the best-fitting model, values of AIC and BIC values were considered, where lower values indicated more parsimonious and better fitting models^61^. Finally, indirect effects were added to the selected Model 5. Because bootstrapping is not available with the MLR estimator, indirect effects were tested by calculating bias-corrected bootstrap (10 000 bootstrap replication samples) 95% confidence interval (CI) with the maximum-likelihood estimator^62,63^.

## Results

### Descriptive statistics and gender-based comparisons

In the current study, 43.8% (*n* = 789) of participants reported no exposure to child maltreatment, while 56.2% (*n* = 1013) reported experiencing at least one form of maltreatment. Exposure to IPV was the most prevalent, at 38.7% of the sample, followed by emotional abuse (27.0%), physical abuse (23.5%), parental neglect (17.5%), and sexual abuse (7.3%).

Table 1 presents descriptive statistics, more particularly percentages, means and standard deviations, as well as correlation coefficients between various forms of maltreatment, alexithymia, and internalizing and externalizing behaviors. The correlations between forms of maltreatment and internalizing/externalizing problems with each constituent of alexithymia can be found as Supplementary Table S1 online. Weak to moderate significant positive associations were found between all forms of maltreatment and alexithymia, as well as with internalizing and externalizing behavior problems. Alexithymia was significantly and moderately positively associated with both internalizing and externalizing behaviors.

**Table 1:** Descriptive statistics and correlations among study variables.

Variable	%/*M* (*SD*)	1	2	3	4	5	6	7
1. Sexual abuse	7.3%	–						
2. Physical abuse	23.5%	0.20***	–					
3. Emotional abuse	27.0%	0.23***	0.44***	–				
4. Parental neglect	17.5%	0.17***	0.34***	0.34***	–			
5. Exposure to IPV	38.7%	0.15***	0.35***	0.34***	0.23***	–		
6. Alexithymia	7.91 (4.83)	0.16***	0.16***	0.22***	0.11***	0.16***	–	
7. Internalizing problems	3.05 (2.83)	0.22***	0.17***	0.31***	0.16***	0.20***	0.50***	–
8. Externalizing problems	1.40 (1.42)	0.18***	0.20***	0.22***	0.20***	0.20***	0.28***	0.38***

***p < 0.001.

Table 2 displays results of chi-square independence tests for maltreatment variables. Overall, post hoc tests using Bonferroni correction (*p* ≤ 0.05) indicated that boys reported significantly lower prevalence of all forms of child maltreatment than gender-diverse individuals, while girls reported significantly lower prevalence than gender-diverse individuals for sexual abuse and parental neglect. Table 3 displays results of the Welch’s ANOVA F test with post hoc group comparisons using Bonferroni tests (*p* ≤ 0.05) for alexithymia and behavior problems. Gender-diverse individuals reported significantly higher scores of alexithymia and internalizing problems than girls, and these groups both reported higher scores than boys. Gender-diverse individuals also reported significantly higher scores of externalizing problems than boys.

**Table 2:** Distribution of child maltreatment forms by gender.

Presence of maltreatment	Boys % (*n*)	Girls % (*n*)	Gender-diverse individuals % (*n*)	χ^2^	*p*
Sexual abuse	3.1 (31)^a^	12.1 (92)^b^	34.6 (9)^c^	79.88	< 0.001
Physical abuse	21.1 (211)^a^	25.7 (195)^a,b^	45.8 (11)^b^	11.74	0.003
Emotional abuse	20.6 (206)^a^	34.0 (259)^b^	70.8 (17)^b^	62.91	< 0.001
Parental neglect	16.3 (163)^a^	18.0 (137)^a^	45.8 (11)^b^	14.54	< 0.001
Exposure to IPV	35.2 (352)^a^	42.6 (325)^b^	62.5 (15)^b^	15.88	< 0.001

Results of chi-square independence tests for maltreatment variables.

^a–c^Groups sharing a common subscript are not statistically significantly different at an alpha level of 0.05 according to the Bonferroni correction procedure.

**Table 3:** Comparisons of boys, girls, and gender-diverse groups of adolescents regarding alexithymia and behavior problems.

	Boys *M* (*SD*)	Girls *M* (*SD*)	Gender-diverse individuals *M* (*SD*)	Welch’s ANOVA F test		
				F	*p*	ω^2^
Alexithymia	6.64 (4.59)^a^	9.41 (4.65)^b^	11.96 (4.37)^c^	87.22	< 0.001	0.09
Internalizing problems	2.0 (2.28)^a^	4.33 (2.89)^b^	5.75 (3)^c^	177.99	< 0.001	0.18
Externalizing problems	1.38 (1.45)^a^	1.38 (1.31)^a^	3.08 (2.32)^b^	6.39	0.003	0.02

Results of the Welch’s ANOVA F test with post hoc group comparisons using Bonferroni tests for alexithymia and behavior problems.

^a–c^Groups sharing a common subscript are not statistically significantly different at an alpha level of 0.05 according to the Bonferroni correction procedure.

### Mediation analysis

All estimated models showed excellent fit (Table 4). In examination of whether hypothesized associations between various forms of maltreatment, alexithymia, and internalizing and externalizing behaviors differed across boys, girls, and gender-diverse individuals, the constrained Model 4 was compared to the unconstrained Model 3. The corrected chi-square was significant (Δχ^2^ = 40.072, *p* = 0.038), and changes in CFI veered from the acceptable range (ΔCFI = 0.015). However, some changes in model fit indices remained in the acceptable range (ΔTLI = 0.008; ΔRMSEA = 0.005), suggesting that the model was gender invariant. Therefore, considering the lower AIC and BIC values, the results of Model 5 (same as Model 2, adding indirect effects) are reported, where all nonsignificant paths were removed, utilizing the total sample and accounting for indirect effects.

**Table 4:** Examination of associations between various forms of child maltreatment, alexithymia, and internalizing and externalizing behavior problems.

Models	AIC	BIC	χ^2 (df)^	CFI	TLI	RMSEA	90% CI
Model 1: Fully saturated model (total sample)	31,943.366	32,185.219	0.000 (0)	1.000	1.000	0.000	0.000–0.000
Model 2: Nonsignificant paths trimmed in the model	31,940.358	32,160.224	4.896 (4)	0.999	0.996	0.011	0.000–0.039
Model 3: Same as Model 2, grouping by gender	30,978.145	31,637.477	16.254 (12)	0.995	0.979	0.024	0.000–0.051
Model 4: Same as Model 3, paths constrained to equality between boys, girls, and gender-diverse individuals	30,968.362	31,484.838	56.550 (38)	0.980	0.971	0.029	0.010–0.043
Model 5: Same as Model 2, adding indirect effects	31,940.358	32,160.224	4.896 (4)	0.999	0.996	0.011	0.000–0.039

*AIC* Akaike information criterion, *BIC* Bayesian information criterion, *χ2* Chi-Square, *df* Degrees of freedom, *CFI* Comparative fit index, *TLI* Tucker–Lewis index, *RMSEA* Root-mean-square error of approximation, *90% CI* 90% confidence interval of the RMSEA.

**Figure 1:**
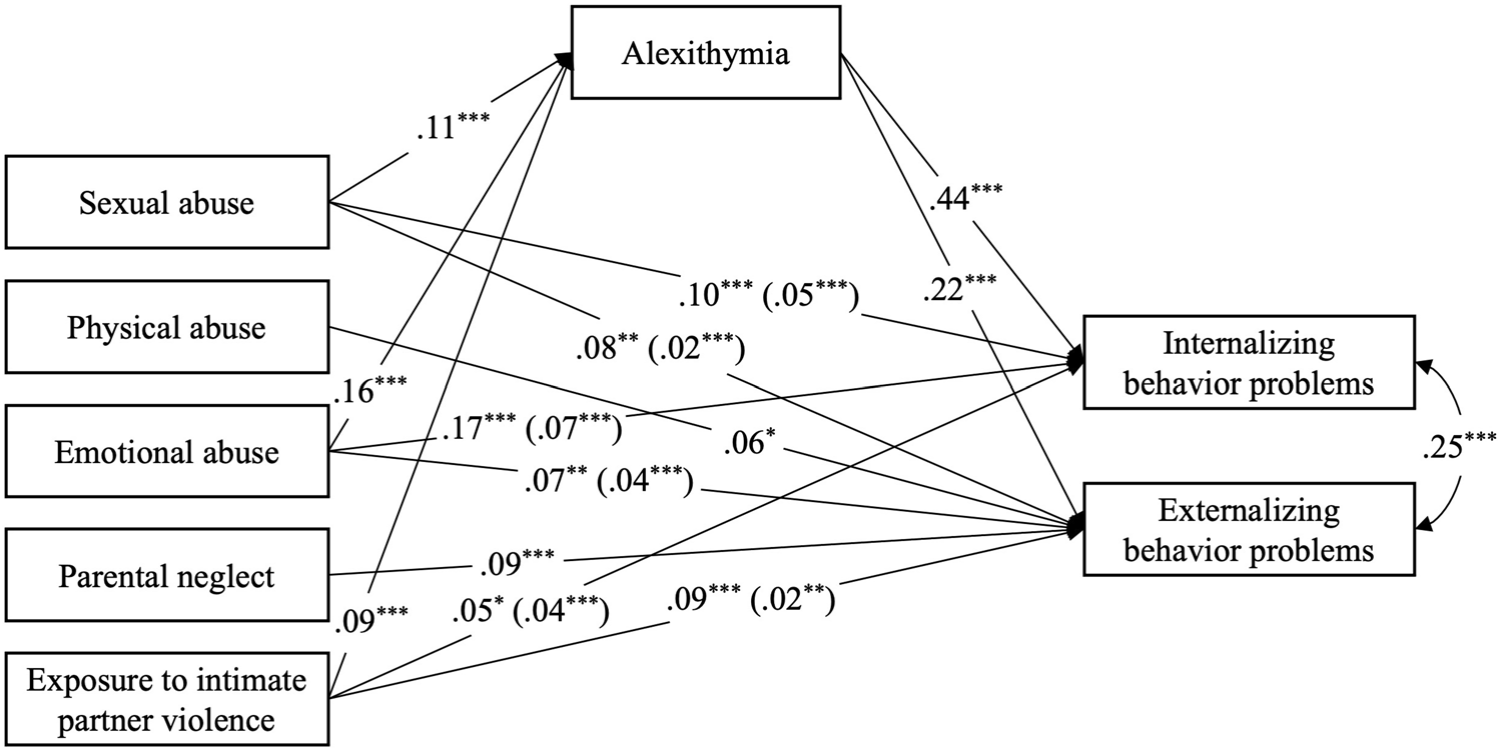
Mediation analysis relating various forms of child maltreatment, alexithymia, and internalizing and externalizing behavior problems. Coefficients in brackets represent indirect effects through alexithymia. ^*^p < 0.05, ^**^p < 0.01, ^***^p < 0.001.

Figure 1 depicts mediation model results, including bootstrapped indirect effects. Results revealed significant direct effects between various forms of child maltreatment and behavior problems. Sexual abuse, β= 0.100; *p* ≤ 0.001 and β  = 0.084; *p* = 0.004, emotional abuse, β= 0.170; *p* ≤ 0.001 and β = 0.071; *p* = 0.007, and exposure to IPV, β  = 0.053; *p* = 0.015 and β= 0.090; *p* ≤ 0.001, were significantly and positively associated with both internalizing and externalizing behaviors. Physical abuse, β = 0.063; *p* = 0.017, and parental neglect, β = 0.094; *p* ≤ 0.001, were associated only with externalizing behaviors. Moreover, indirect effects were found in associations between three forms of maltreatment and behavior problems through alexithymia. Specifically, exposure to sexual abuse, *β* = 0.049, 95% bootstrap CI = [0.028, 0.070] and *β* = 0.024, 95% bootstrap CI = [0.014, 0.036], emotional abuse, *β* = 0.071, 95% bootstrap CI = [0.051, 0.094] and *β* = 0.035, 95% bootstrap CI = [0.023, 0.049], and exposure to IPV, *β* = 0.039, 95% bootstrap CI = [0.018, 0.061] and *β* = 0.019, 95% bootstrap CI = [0.009, 0.032], were associated with higher levels of alexithymia, which in turn related to more internalizing and externalizing behavior problems. The model explained 31% of internalizing behavior problems’ variance and 14% of externalizing behavior problems’ variance.

## Discussion

By examining alexithymia’s mediating role and potential gender differences, this study investigated associations among various forms of child maltreatment (i.e., sexual, physical, and emotional abuse; parental neglect; and exposure to IPV) and adolescents’ internalizing and externalizing problems. Results showed that sexual abuse, emotional abuse, and exposure to IPV were directly associated with greater alexithymia and both internalizing and externalizing problems, while physical abuse and parental neglect were related only to greater externalizing problems. Overall, our findings suggest that adolescents who were childhood victims of sexual abuse, emotional abuse, and exposure to IPV experience higher levels of alexithymia, which are in turn associated with more internalizing and externalizing behaviors, regardless of gender identity. Therefore, these results support alexithymia’s mediating role in associations between three forms of maltreatment and behavior problems in adolescence.

The hypothesis that various forms of child maltreatment would be directly and positively associated with behavior problems was partially supported. Indeed, three forms of child maltreatment (i.e., sexual abuse, emotional abuse, and exposure to IPV) were directly linked to both types of behavioral problems. These findings align with previous work suggesting that victims of child sexual abuse were more likely than non-victims and those maltreated other than sexually to experience internalizing and externalizing problems during adolescence^4^. Regarding emotional abuse, this study’s results mirrored those of previous work on adults and adolescents for both internalizing^20,64,65^ and externalizing behaviors^64,66^. Moreover, meta-analysis found moderate associations between childhood exposure to IPV and both internalizing and externalizing behaviors in adolescence^67^, these results have been corroborated by other studies among children^68^ and adolescents^5^. Although previous studies have found that neglect^15,69^ and physical abuse^15,64^ were associated with both types of behavioral difficulties and although we found these associations at a correlational level, they were associated only with externalizing problems in the integrated model. This may be related to the ways in which child maltreatment forms were measured in our study relative to other studies (e.g., the items and the scale used). Nonetheless, according to Zahn-Waxler et al.^70^, while internalizing problems do exist in childhood, they tend to become more pronounced during adolescence, whereas externalizing problems typically manifest earlier in childhood. Therefore, in our sample, it is possible that physically abused or neglected adolescents had not yet exhibited significant internalizing problems, which may emerge over the longer term. Possibly, some forms of child maltreatment are more deleterious than others. For example, previous studies found stronger relationships between emotional abuse^20,66^ or sexual abuse^4^ and behavior problems when compared to other forms of maltreatment. Such findings suggest that different forms of maltreatment have specific effects on internalizing and externalizing behavior problems, thus demonstrating adverse childhood experiences’ complexity and impact on young lives. These findings also demonstrate the importance of future investigations into separate types of behavior problems.

Regarding this study’s second hypothesis, our findings suggest that adolescents who reported childhood sexual abuse, emotional abuse, or exposure to IPV presented higher levels of alexithymia, which, in turn, were associated with more internalizing and externalizing behavior problems. These findings align with previous childhood studies that have demonstrated the mediating role of alexithymia between sexual abuse^29^ and exposure to IPV and both internalizing and externalizing problems^30^. Related research has also demonstrated that emotional abuse and neglect were associated with alexithymia, which, in turn, contributed to emergence of somatic symptoms (i.e., internalizing problems) in adolescents’ major depressive disorders^71^. Although previous studies have shown physical abuse and parental neglect to have negative effects on development of emotion regulation skills^9^, the present study did not show alexithymia to mediate relationships between these two forms of maltreatment and behavioral problems. Possibly the single item measuring physical abuse and the two items measuring parental neglect were not detailed enough to capture all manifestations of these maltreatment forms. Not only do these results provide new insight about which forms of maltreatment are associated with which behavioral problems, they also add a new dimension to the consequences of childhood maltreatment during adolescence—a developmental period characterized by the development of emotion regulation skills^39^. In various populations, moreover, emotional regulation problems such as alexithymia are associated with increased risk of internalizing and externalizing problems following maltreatment^22,29,30,36^. Research suggests that childhood maltreatment is associated with significant risk of developing alexithymia^72^ due to the lack of caregiver modeling for acquisition of emotion identification and description skills and of safety in expressing emotions^73^. Deficits in these skills, characteristic of alexithymia, would in turn generate more internalizing (e.g., depression, anxiety, sense of loneliness)^20^ and externalizing behavior problems (e.g., hyperactivity, conflicts with peers, conduct problems)^25^. Thus, alexithymia appears to be a key factor and a relevant construct in understanding development of behavior problems among adolescents who have experienced childhood maltreatment (e.g., by identifying difficulties specific to alexithymia, such as differentiating and expressing emotions)^21^.

Preliminary analyses suggested that girls were exposed to greater child maltreatment (i.e., sexual abuse, emotional abuse, and IPV) and reported higher levels of alexithymia and internalizing problems compared with boys. Moreover, compared to boys, gender-diverse individuals were more exposed to all forms of maltreatment and reported higher levels of alexithymia and behavioral problems; compared to girls, these individuals were more exposed to sexual abuse and parental neglect and presented higher levels of alexithymia and internalizing behaviors. These results concur with previous reviews and studies on gender-diverse individuals, suggesting that they experience higher rates of child maltreatment^74–76^, internalizing problems (e.g., depression, suicidal ideation)^75^, psychological distress^77,78^, and mental health concerns^75,77,78^ than their peers. According to minority stress theory^79,80^, gender-diverse individuals’ exposure to child maltreatment can contribute to this population’s observed mental health disparities and variations in psychological distress^78^. Surprisingly, however, our results also revealed that associations between child maltreatment, alexithymia, and behavioral problems were not stronger among gender-diverse adolescents (and not between boys and girls) compared to boys and girls in the integrated model. Therefore, our sample’s absence of significant differences might indicate that alexithymia’s mediating role in relationships between various forms of child maltreatment and behavioral outcomes among boys, girls, and gender-diverse adolescents is similar. Among girls and gender-diverse adolescents, gender disparities perhaps contribute to greater risk of child maltreatment, alexithymia, and behavioral problems, but more importantly, gender disparities do not shape the associations between these constructs.

### Strengths, limitations, and future studies

Results from the current study must be interpreted in light of some limitations. The cross-sectional design limits the ability to draw inferences about directionality and causality among variables. Besides that, results might have been influenced by social desirability in the use of self-report measures. In addition, although all measures were validated, they were not necessarily validated with the population constituting our sample of French, predominantly Caucasian adolescents; this also limits the findings’ generalizability. In addition, the low internal reliability of the conduct problem scale might have underestimated externalizing behavior. Notably, this study did not explicitly investigate pre-existing traits that could also contribute to these outcomes. For instance, individuals with autism spectrum disorder tend to exhibit higher levels of alexithymia^81^, which could independently influence their internalizing and externalizing behavior patterns. Moreover, neurodevelopmental disorders are often associated with experiences of abuse and may exacerbate internalizing and externalizing behavioral problems^82,83^.

Despite these limitations, our study addressed previous gaps in the literature ^28^ by focusing on a sample of adolescents and by examining alexithymia as a mechanism in associations between various forms of child maltreatment and adolescents’ behavior problems. Other strengths include use of a large sample and robust, appropriate statistical techniques. Furthermore, great strengths include recruitment of a socio-demographically diverse sample and inclusion of gender-diverse individuals.

Future studies should use longitudinal designs to examine directionality of associations between various forms of child maltreatment, alexithymia, and internalizing and externalizing behavior problems among adolescents. To further understand the role of alexithymia and to gain insight into its nuanced associations with psychological outcomes among adolescents, studies could explore the different constituents of alexithymia (i.e., difficulty identifying and describing feelings). Nonetheless, given this study’s partial mediational effect, future research should investigate other mechanisms implicated in associations between various forms of child maltreatment and behavior problems (e.g., attachment styles)^84^. Because co-occurrence of different maltreatment forms has been associated with greater risk of experiencing a wide range of internalizing and externalizing problems^5,48^, future studies should investigate such influences. Finally, the influence of these pre-existing traits should be further investigated to better understand their potential contribution to the observed outcomes.

## Conclusion

This study supports existing literature on negative effects of different forms of child maltreatment and negative outcomes in adolescence. Results suggest that adolescents with childhood experience of sexual or emotional abuse, as well as exposure to IPV, are more likely to exhibit internalizing and externalizing problems. Findings also expand knowledge by identifying alexithymia as a mechanism explaining associations between child maltreatment and behavior problems in adolescence. Thus, the study highlights the importance of interventions to enhance emotional awareness and expression in adolescents with a childhood history of maltreatment.

### Supplementary Information

Supplementary Information.

## Data Availability

The data presented in this study are available on request from the corresponding author. The data, which were used under license for the current study, are therefore not publicly available due to ethical restrictions.
