# Author Correction: Alexithymia as a mediator of the associations between child maltreatment and internalizing and externalizing behaviors in adolescence

**DOI:** 10.1038/s41598-024-59645-9

**Published:** 2024-04-16

**Authors:** Catherine Hamel, Christopher Rodrigue, Camille Clermont, Martine Hébert, Linda Paquette, Jacinthe Dion

**Affiliations:** 1 Département de Psychologie, Université de Montréal, Montréal, H2V 2S9 Canada; 2 Research Centre On Intimate Relationship Problems and Sexual Abuse (CRIPCAS), Université de Montréal, Montréal, H2V 2S9 Canada; 3 École de Psychologie, Université Laval, Québec, G1V 0A6 Canada; 4 Département de Sexologie, Université du Québec À Montréal, Montréal, H2L 4Y2 Canada; 5 Département Des Sciences de La Santé, Université du Québec À Chicoutimi, Chicoutimi, G7H 2B1 Canada; 6 Département de Psychologie, Université du Québec À Trois-Rivières, Trois-Rivières, G9A 5H7 Canada

Correction to: *Scientific Reports* 10.1038/s41598-024-56909-2, published online 16 March 2024

The original version of this Article contained errors in the Affiliations.

Martine Hébert was incorrectly affiliated with ‘Département Des Sciences de La Santé, Université du Québec À Chicoutimi, Chicoutimi G7H 2B1, Canada’.

The correct affiliations for Martine Hébert are listed below.

Research Centre On Intimate Relationship Problems and Sexual Abuse (CRIPCAS), Université de Montréal, Montréal H2V 2S9, Canada.

Département de Sexologie, Université du Québec À Montréal, Montréal H2L 4Y2, Canada.

Additionally, Linda Paquette was incorrectly affiliated with ‘Département de Sexologie, Université du Québec À Montréal, Montréal H2L 4Y2, Canada’.

The correct affiliation for Linda Paquette is listed below.

Département Des Sciences de La Santé, Université du Québec À Chicoutimi, Chicoutimi G7H 2B1, Canada.

The original Article has been corrected.

