# Supplementary material for: Alexithymia as a mediator of the associations between child maltreatment and internalizing and externalizing behaviors in adolescence
Source: Sci Rep. 2024 Mar 16;14:6359. doi: 10.1038/s41598-024-56909-2 (PMC10944459; doi:10.1038/s41598-024-56909-2)
Supplement: Supplementary file 1 — Supplementary Information. [file 41598_2024_56909_MOESM1_ESM.pdf]

Alexithymia as a mediator of the associations between child maltreatment and internalizing and externalizing behaviors in adolescence

Catherine Hamel[1,2*], Christopher Rodrigue[2,3], Camille Clermont[2,3], Martine Hébert[2,5], Linda Paquette[4], and Jacinthe Dion[2,6]

[1]Département de Psychologie, Université de Montréal, Montréal, H2V 2S9, Canada
[2]Research Centre on Intimate Relationship Problems and Sexual Abuse (CRIPCAS), Université de Montréal, Montréal, H2V 2S9, Canada
[3]École de Psychologie, Université Laval, Québec, G1V 0A6, Canada
[4]Département de Sexologie, Université du Québec à Montréal, Montréal, H2L 4Y2, Canada
[5]Département des Sciences de la Santé, Université du Québec à Chicoutimi, Chicoutimi, G7H 2B1, Canada
[6]Département de Psychologie, Université du Québec à Trois-Rivières, Trois-Rivières, G9A 5H7, Canada
[*]catherine.hamel.6@umontreal.ca

Variable	Difficulty identifying feelings	Difficulty describing feelings
Sexual abuse	0.17^{***}	0.09^{***}
Physical abuse	0.16^{***}	0.10^{***}
Emotional abuse	0.22^{***}	0.14^{***}
Parental neglect	0.11^{***}	0.07^{**}
Exposure to IPV	0.17^{***}	0.09^{***}
Internalizing problems	0.51^{***}	0.33^{***}
Externalizing problems	0.28^{***}	0.18^{***}
M (SD)	5.55 (3.92)	2.36 (1.32)

Supplementary Table S1. Examination of associations between each constituent of alexithymia, various forms of child maltreatment, and behavior problems. $^{**}p < 0.01$, $^{***}p < 0.001$
